# Baseline lymphocyte and cholinesterase levels may be the predictors of chronic herbal drug–induced liver injury

**DOI:** 10.3389/fphar.2022.962480

**Published:** 2022-08-05

**Authors:** Zhan Zeng, Wei Yi, Jian-Ping Dong, Qi-Qi Chen, Fang-Fang Sun, Hui-Hui Lu, Yan-Jie Lin, Xiao-Yue Bi, Liu Yang, Yao Lu, Lu Zhang, Ming-Hui Li, Yao Xie

**Affiliations:** ^1^ Department of Hepatology Division 2, Peking University Ditan Teaching Hospital, Beijing, China; ^2^ Department of Gynecology and Obstetrics, Beijing Ditan Hospital, Capital Medical University, Beijing, China; ^3^ Department of Infectious Diseases, Haidian Hospital, Beijing Haidian Section of Peking University Third Hospital, Beijing, China; ^4^ Department of Hepatology Division 2, Beijing Ditan Hospital, Capital Medical University, Beijing, China

**Keywords:** drug-induced liver injury, herbal medicine, chronicity, predictors, hepatitis

## Abstract

**Objective:** To investigate the factors influencing the chronicity of drug-induced liver injury (DILI) caused by Chinese herbal medicine.

**Methods:** Patients with DILI diagnosed by using the RUCAM score were enrolled retrospectively. The subjects were patients with DILI induced by taking Chinese herbal medicine and were followed up for 48 weeks. These patients were divided into a cure group and a chronic group. The biochemical indicators were monitored at baseline and every 3 months. Logistic regression was used to analyze the risk factors of DILI chronicity. The ROC (receiver operator characteristic) curve was used to analyze the diagnostic efficiency of each factor.

**Results:** A total of 420 patients with DILI were enrolled; 122 of them were caused by Chinese herbal medicine, 70.5% (86/122) of them were female, chronic group 31.2% (39/122), and cure group 68.0% (83/122); cholinesterase (ChE) in the chronic group was lower than that in the cure group (5467.10 ± 2010.40 U/L vs. 6248.52 ± 1901.78 U/L, *p* = 0.04, t = 2.078). There was no significant difference in the age between cured patients and chronic patients (*p* = 0.156, Z = −1.417). There was no significant difference between the prognosis of different genders (*p* = 0.521, Z = −0.639). The logistic regression analysis showed that baseline lymphocyte (OR = 0.429, 95%CI = 0.205–0.898, *p* = 0.025) and cholinesterase (OR = 0.088, 95%CI = 0.008–0.994, *p* = 0.049) were independent risk factors of drug-induced chronicity.

**Conclusion:** Baseline lymphocyte and cholinesterase may be the predictive factors for the chronicity of Chinese herbal medicine–induced liver injury.

## 1 Introduction

Drug-induced liver injury (DILI) is one of the most common adverse drug reactions. The incidence of DILI in China is about 28.30/100000 (Shen et al., 2019), which is higher than that in European and American countries, and the common causes include the use of traditional Chinese medicine (TCM), herb and dietary supplements (HDS), and antituberculosis drugs (antituberculosis medications). (Shen et al., 2019). The most common manifestation of DILI is acute liver injury. DILI is one of the main causes of acute liver failure in western countries. Most patients recover from acute liver injury with remission of clinical symptoms and normal liver function tests. But a small number of patients with lesions maintained for more than 6 months are called chronic (Chalasani et al., 2008; Yu et al., 2017), and different studies have shown that the proportion of DILI chronicity are between 13% and 18.9% (Chalasani et al., 2008; Fontana et al., 2014; Chalasani et al., 2015; Shen et al., 2019). There are different risk factors for the chronicity of DILI; for example, biliary stasis type and mixed type DILI are more likely to be chronic than hepatocellular type DILI, and drugs for cardiovascular and central nervous system diseases are more likely to lead to chronicity (Andrade et al., 2006; Qiqi et al., 2020). Chinese herbal medicine is one of the main inducements of DILI in China. Up to now, there is no study on the risk factors of chronic DILI caused by Chinese herbal medicine. The purpose of this study is to collect the data of DILI from September 2014 to September 2018 in the Department of Hepatology Division 2, Beijing Ditan Hospital and analyze the risk factors of chronic DILI caused by Chinese herbal medicine.

## 2. Materials and methods

### 2 1 Study object

Patients with an abnormal liver function and clinically diagnosed as DILI by using the RUCAM score in Beijing Ditan Hospital affiliated to Capital Medical University from September 2014 to September 2018 were enrolled retrospectively. In these patients, patients with DILI caused by Chinese herbal medicine were selected as our study object. This study was approved by the Ethical Committee of Beijing Ditan Hospital affiliated to Capital University of Medical Sciences [JDLYZ (2020) (005) -01)]. ClinicalTrials.gov, Number: NCT04302506.

### 2.2 Inclusion and exclusion criteria

#### 2.2.1 Inclusion criteria

1) Having a definite history of taking Chinese herbal medicine and dietary supplements; 2) DILI patients were diagnosed by RUCAM. Diagnostic criteria refer to the RUCAM scale score: score range 0–14 points; > 8 points: highly possible (or certain); 6–8 points: very possible; 3–5 points: possible; 1–2 points: impossible, and 0 points can be excluded. Those with score ≥6 were included in this study (Yu et al., 2017).

#### 2.2.2 Exclusion criteria

1) Complicated with other liver diseases, such as viral hepatitis, alcoholic hepatitis, autoimmune hepatitis, metabolic hepatitis, and non-alcoholic fatty liver disease; 2) complicated with other viral infections, such as the Epstein–Barr virus (EBV), cytomegalovirus (CMV), and human immunodeficiency virus (HIV); 3) mental illness; 4) evidence of liver neoplasms (HCC or AFP >100 μg/L); and 5) Other chronic diseases such as diabetes and hypertension.

### 2.3 Study design

**FIGURE 1:**
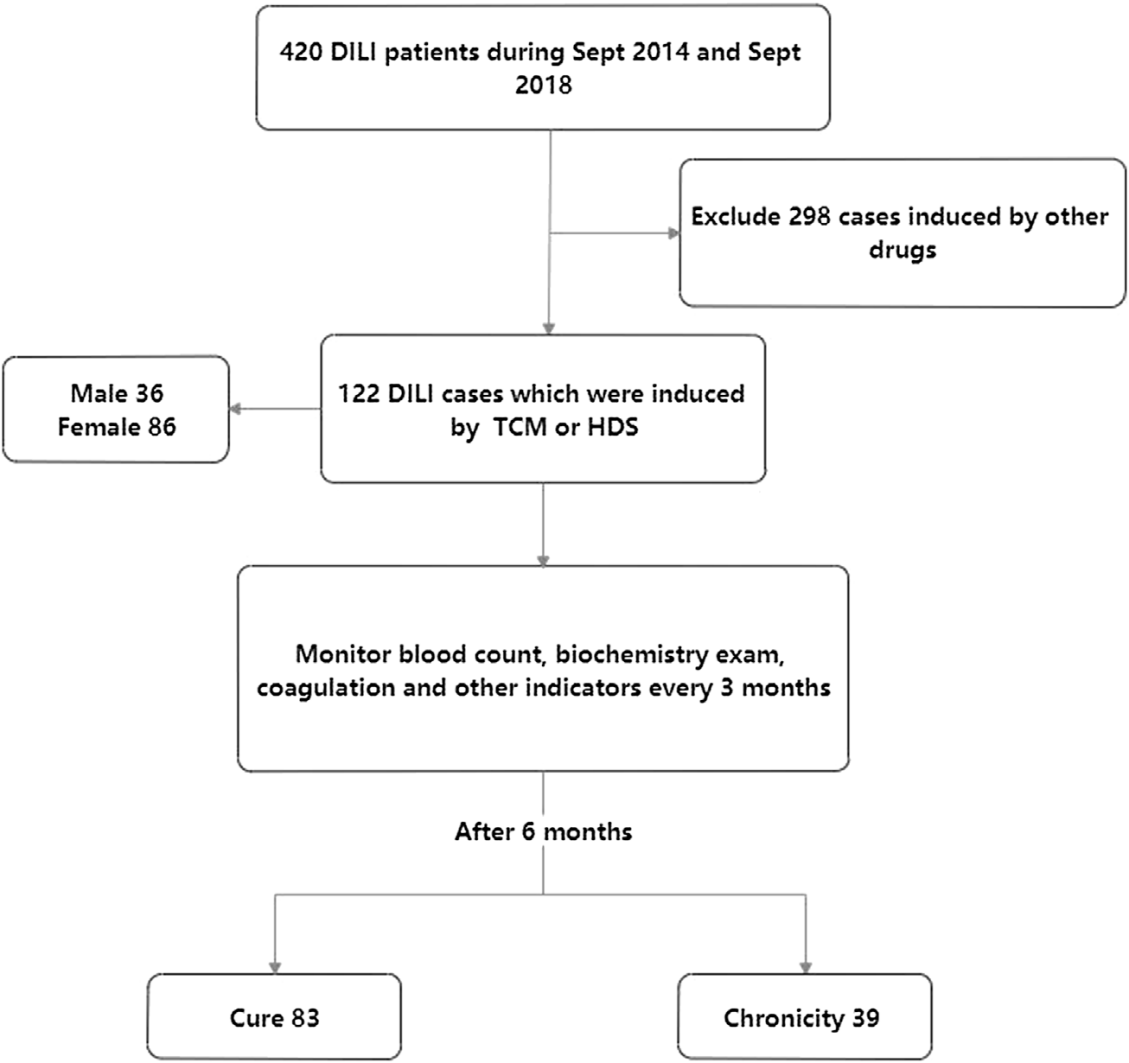
Enrollment of trial participants.

The patients with DILI caused by Chinese herbal medicine from September 2014 to September 2018 as the research objects were enrolled retrospectively and followed for 48 weeks. The liver histopathological data of the patients were collected, including diagnosis, degree of liver inflammation and fibrosis, and whether combined with other factors cause liver pathological changes. Etiology, blood routine, biochemistry, coagulation function, ESR, and thyroid hormone were monitored at baseline and every 3 months. According to the prognosis, the patients were divided into the cure group and the chronic group (Figure 1). Univariate and multivariate logistic regression were used to analyze the risk factors of DILI chronicity. The ROC curve was used to analyze the diagnostic efficacy of each factor.

### 2.4 Definition of chronic drug-induced liver injury

After 6 months of DILI, serum ALT, AST, ALP, or TBil are still abnormal, or there are imaging and histological evidences of portal hypertension or chronic liver injury. If the aforementioned conditions are met, it can be diagnosed as chronic drug-induced liver injury (Yu et al., 2017).

### 2.5 Treatment

Depending on the patient’s symptoms and condition, different combinations of drugs are used to treat. For example, 103 patients were treated with reduced glutathione, 78 patients treated with polyene phosphatidyl choline, 111 patients treated with glycyrrhizin, 26 patients treated with ursodeoxycholic acid, eight patients treated with tiopronin, and 76 patients treated with silymarin.

### 2.6 Baseline indicators

#### 2.6.1 Baseline data

1) Demographic data (age, sex); 2) collection of drug data and use information: mainly the name, dosage, and duration of drugs that may cause liver drug-induced injury before or at the onset of hepatitis; 3) collection of liver histopathological data: collect the liver histopathological data, including diagnosis, degree of liver inflammation and fibrosis, and whether combined with other factors cause liver pathology. 4) The hematological index, including clinical biochemical indicators: alanine aminotransferase (ALT), aspartate aminotransferase (AST), total bilirubin (TBil), direct bilirubin (DBil), ALP (alkaline phosphatase), albumin (ALB), total bile acid (TBA), and cholinesterase (ChE). The blood routine test, coagulation index [prothrombin activity (PTA), international normalized ratio (INR)], erythrocyte sedimentation rate (ESR), thyroid hormone, alpha fetoprotein (AFP), blood glucose, blood lipid, special protein, autoimmune liver disease, and abdominal B ultrasound; and 5) Treatment after diagnosis of DILI.

### 2.7 Observation index every 3 months

The blood routine test, biochemical test, and coagulation function of patients were recorded every 3 months from the baseline until 48 weeks.

### 2.8 Statistical analysis

SPSS 19.0 and GraphPad Prism 5 were used for statistical analysis. The measurement data of non-normal distribution (age, ALT, AST, TBil, DBil, ALP, and TBA) were expressed as M (P25, p75). The comparison between groups was performed with the non-parametric Mann–Whitney U rank sum test; counting data (proportion of males, proportion of chronicity) were expressed as cases and percentages and compared with χ^2^ test. Binary logistic regression was used to analyze the predictors of DILI outcome. All tests were two-sided ; *p* < 0.05 as a statistically significant difference.

## 3 Results

### 3.1 General condition of patients

A total of 420 patients were diagnosed with DILI by using the RUCAM score, of which 122 cases were caused by Chinese herbal medicine, accounting for about 29.1%; Of the 122 patients, 29.5% (36/122) were male and 70.5% (86/122) were female; 68.0% (83/122) were cured, 32.0% (39/122) were chronic, and the age was 49 (34, 56). All patients did not appear death and other serious adverse consequences.

### 3.2 Implicated agents and treatment

Among the 122 patients, 88 cases had the history of taking Chinese herbal medicine and seven of them had been taking Chinese herbal medicine for a long time (specific drug ingredients was unclear). In addition, 34 of the 122 patients took Chinese patent medicines (14 cases of Lianhua Qingwen capsule, three cases of LiuweiDihuang pill, three cases of HuoXiangZhengQiShui, three cases of NiuHuangJieDu pill, three cases of LingZhiYiShou capsule, one case of JiaWeiXiaoYaoWan, one case of YangXueRongJinWan, and six cases of ZiBuShengFa pill). The reasons for taking traditional Chinese medicine included weight loss, stomach disease treatment, hair loss treatment, insomnia, and so on.

There were no significant differences between the therapeutic agents used in the different prognostic subgroups, including reduced glutathione (***χ*** ^***2***^ = 0.246, *p* = 0.620), polyene phosphatidyl choline (***χ*** ^***2***^ = 0.143, *p* = 0.706), glycyrrhizin (***χ*** ^***2***^ = 0.123, *p* = 0.726), ursodeoxycholic acid [***χ*** (Yu et al., 2017) = 0.107, *p* = 0.744], tiopronin (***χ*** ^***2***^ = 1.49, *p* = 0.222), and silymarin (***χ*** ^***2***^ = 1.17, *p* = 0.279).

### 3.3 Baseline data

Baseline in the cured group: ALT = 336 (108.9, 767), AST = 190.3 (74.9, 401.8), Tbil = 22 (14, 96), Dbil = 10.4 (5.9, 71.9), CHE = 6248.52 ± 1901.78, LYM = 1.56 (1.310, 1.995), PTA = 92.5 (80, 102.3), and AFP = 7.00 (3.55, 24.00). Baseline in the chronic group: ALT = 335.3 (154.5,643), AST = 223 (135.5, 404), Tbil = 45.9 (18, 124.2), Dbil = 32 (9.8, 102.6), CHE = 5467.10 ± 2010.40, LYM = 1.35 (1.12, 1.81), PTA = 87.0 (74.0, 100.0), and AFP = 7.90 (4.00, 24.28) (Table 1). The baseline cholinesterase in the chronic group was significantly lower than that in the cure group (*p* = 0.04, t = 2.078), while no significant difference was found in other indexes. The mean thickness of the spleen in the cure group was 36.23 ± 7.11 mm, and the mean thickness of the spleen in the chronic group was 36.46 ± 7.24 mm. There was no significant difference between the two groups (*p* = 0.878, t = −0.154). No patients were diagnosed with liver cancer, and six patients were diagnosed with liver cirrhosis.

**TABLE 1:** Comparison of selected characteristics of baseline due to different prognosis.

Characteristics	Cure	Chronicity	*p value*	*z/t/χ*
ALT (U/L)	336 (108.9, 767)	335.3 (154.5, 643)	0.725	−0.351
AST (U/L)	190.3 (74.9, 401.8)	223 (135.5, 404)	0.531	−0.626
Tbil (umol/l)	22 (14, 96)	45.9 (18, 124.2)	0.111	−1.592
Dbil (umol/l)	10.4 (5.9, 71.9)	32 (9.8, 102.6)	0.105	−1.622
ALP (u/l)	118 (88, 163.6)	140.9 (104, 163)	0.098	−1.653
TBA (umol/L]	28 (9.4, 163.6)	35 (19, 160)	0.444	−0.765
ALB (g/L)	38.38 ± 5.11	38.07 ± 3.58	0.694	0.395
GLO (g/L)	29 (26, 31.7)	30.9 (27, 34)	0.071	−1.806
GGT (U/L)	123 (75, 252)	168 (75, 244)	0.600	−0.524
CHE (U/L)	6248.52 ± 1901.78	5467.10 ± 2010.40	0.040	2.078
WBC (×10^9^/L)	4.60 (3.82, 5.81)	4.79 (3.92, 5.73)	0.704	−0.380
Hb (U/L)	129.05 ± 17.88	128.50 ± 14.84	0.870	0.164
PLT (×10^9^/L)	189.33 ± 69.95	208.95 ± 71.69	0.160	0.155
LYM (×10^9^/L)	1.56 (1.310, 1.995)	1.35 (1.12, 1.81)	0.066	−1.835
PTA (S)	92.5 (80, 102.3)	87.0 (74.0, 100.0)	0.305	−1.026
INR	1.06 (1.00, 1.20)	1.05 (0.98, 1.16)	0.188	−1.315
T3 (ng/ml)	1.04 ± 0.32	0.912 ± 0.06	0.062	1.89
T4 (ug/dl)	10.1 ± 3.47	9.07 ± 3.48	0.193	1.31
TSH (uIU/ml)	1.67 (1.13, 2.77)	1.28 (1.08, 2.44)	0.181	−1.338
FT3 (pg/ml)	2.42 ± 0.54	2.31 ± 0.49	0.342	0.96
FT4 (ng/dl)	1.07 (0.96, 1.22)	1.09 (0.99, 1.26)	0.754	−0.314
ESR (×10^9^/L)	7.00 (3.75, 12.50)	7.5 (2.0, 16.5)	0.692	−0.396
AFP (ng/ml)	7.00 (3.55, 24.00)	7.90 (4.00, 24.28)	0.943	−0.071
Necroinflammatory activity	/	/	0.777	0.506
Mild	67.5%	66.7%	/	/
Moderate	31.3%	33.3%	/	/
Severe	1.2%	0%	/	/
Fibrosis	/	/	0.482	2.463
No fibrosis	21.7%	30.8%	/	/
Mild	71.1%	66.7%	/	/
Moderate	3.6%	2.6%	/	/
Severe	3.6%	0%	/	/
Interface inflammation	/	/	0.722	1.328
No interface inflammation	78.3%	79.5%	/	/
Mild	14.5%	17.9%	/	/
Moderate	6.0%	2.6%	/	/
Severe	1.2%	0%	/	/
Bile duct injury	6.0%	12.8%	0.356	0.851
Ductopenia	1.2%	0%	0.491	0.474
Bile duct hyperplasia	72.3%	71.8%	0.955	0.003

### 3.4 Pathological results of liver tissue

Comparison of pathological results in patients with different prognoses revealed no significant differences in the incidence of necroinflammatory activity (***χ*** ^***2***^ = 0.506, *p* = 0.777), fibrosis (***χ*** ^***2***^ = 2.463, *p* = 0.482), interface inflammation (***χ*** ^***2***^ = 1.328, *p* = 0.722), bile duct injury (***χ*** ^***2***^ = 0.851, *p* = 0.356), ductopenia (***χ*** ^***2***^ = 0.474, *p* = 0.491), and bile duct hyperplasia (***χ*** ^***2***^ = 0.003, *p* = 0.955) between the two groups (Table 1). In addition, 57.4% (70/122) of DILI patients were hepatocellular injury type, 13.9% (17/122) were cholestatic type, and 28.7% (35/122) were mixed type.

### 3.5 Characteristics of patients at 6-month follow-up

Baseline in the cure group: ALT = 26.5 (18.4, 51.1), AST = 33.7 (19.4, 50.7), Tbil = 10.6 (8.8, 21.1), Dbil = 3.9 (2.2, 7.9), ChE = (7898.9 ± 2061.7), LYM = 1.92 (1.26, 2.37), ALB = (44.6 ± 5.8), ALP = 80.0 (66.1, 114.0), and TBA = 6.2 (3.6, 14). Baseline in the chronic group: ALT = 65.9 (49.4, 119.4), AST = 52.2 (33.3, 130), Tbil = 12.1 (8.3, 20.0), Dbil = 4.7 (2.2, 11.0), ChE = 6383.7 ± 2491.1, LYM = 1.47 (1.34, 2.13), ALB = (40.7 ± 5.6), ALP = 137.2 (73.6, 157.9), and TBA = 20.9 (6.2, 53.8). There were significant differences in ALT, AST, ALP, TBA, ALB, and ChE (*p* < 0.05) (Table 2).

**TABLE 2:** Comparison of selected characteristics of 6 months due to different prognosis.

Characteristics	Cure	Chronicity	*p* value	*z/t*
ALT (U/L)	26.5 (18.4, 51.1)	65.9 (49.4, 119.4)	0.000012	−4.387
AST (U/L)	33.7 (19.4, 50.7)	52.2 (33.3, 130)	0.002	−3.052
Tbil (umol/l)	10.6 (8.8, 21.1)	12.1 (8.3, 20.0)	0.837	−0.205
Dbil (umol/l)	3.9 (2.2, 7.9)	4.7 (2.2, 11.0)	0.497	−0.679
ALP (u/l)	80.0 (66.1, 114.0)	137.2 (73.6, 157.9)	0.003	−2.924
TBA (umol/L]	6.2 (3.6, 14)	20.9 (6.2, 53.8)	0.004	−2.908
ALB (g/L)	44.6 ± 5.8	40.7 ± 5.6	0.003	3.087
CHE (U/L)	7898.9 ± 2061.7	6383.7 ± 2491.1	0.002	3.113
LYM (×10^9^/L)	1.92 (1.26, 2.37)	1.47 (1.34, 2.13)	0.766	−0.298

### 3.6 Predictive analysis

We screened variables with *p* < 0.05 for multivariate analysis by univariate logistic regression. Also, logistic univariate and multivariate regression analyses showed that the baseline LYM count (OR = 0.429, 95%CI = 0.205–0.898, *p* = 0.025) and ChE (OR = 0.088, 95%CI = 0.008–0.994, *p* = 0.049) were independent risk factors for TCM-induced chronicity. Necroinflammatory activity, fibrosis, biliary duct injury, interface inflammation, ductopenia, and bile duct hyperplasia were analyzed by univariate logistic regression, and the results showed that none of these factors was a risk factor (*p* < 0.05) (Table 3).

**TABLE 3:** Baseline data univariate logistic regression analysis.

Variables	*OR*	*p*	95%*CI*
ALT	1.000	0.295	0.999–1.000
AST	1.000	0.808	0.999–1.002
TBIL	1.002	0.433	0.998–1.006
DBIL	1.003	0.346	0.997–1.008
ALP	1.004	0.080	0.999–1.009
GGT	1.000	0.645	0.998–1.002
GLO	1.043	0.254	0.970–1.121
TBA	1.000	0.968	0.996–1.004
ChE	0.088	0.049	0.008–0.994
PTA	0.997	0.752	0.981–1.014
INR	0.082	0.083	0.005–1.393
WBC	1.109	0.270	0.923–1.334
NEU	0.984	0.848	0.837–1.157
LYM	0.429	0.025	0.205–0.898
PLT	1.004	0.158	0.998–1.010
AFP	0.999	0.857	0.984–1.013
T3	0.228	0.066	0.047–1.102
T4	0.917	0.192	0.804–1.045
TSH	0.725	0.094	0.498–1.056
FT3	0.656	0.338	0.277–1.554
FT4	1.128	0.916	0.121–10.526
Necroinflammatory activity	/	/	/
Mild	ref	/	/
Moderate	0.074	0.858	0.478–2.426
Fibrosis	/	/	/
No fibrosis	ref	/	/
Mild	−0.414	0.348	0.279–1.570
Moderate	−0.693	0.568	0.046–5.390
Interface inflammation	/	/	/
No Interface inflammation	ref	/	/
Mild	0.201	0.700	0.439–3.411
Moderate	0.869	0.437	0.047–3.744
No bile duct injury	ref	/	/
Bile duct injury	0.830	0.212	0.623–8.446
No bile duct hyperplasia	ref	/	/
Bile duct hyperplasia	−0.025	0.955	0.418–2.276

**FIGURE 2:**
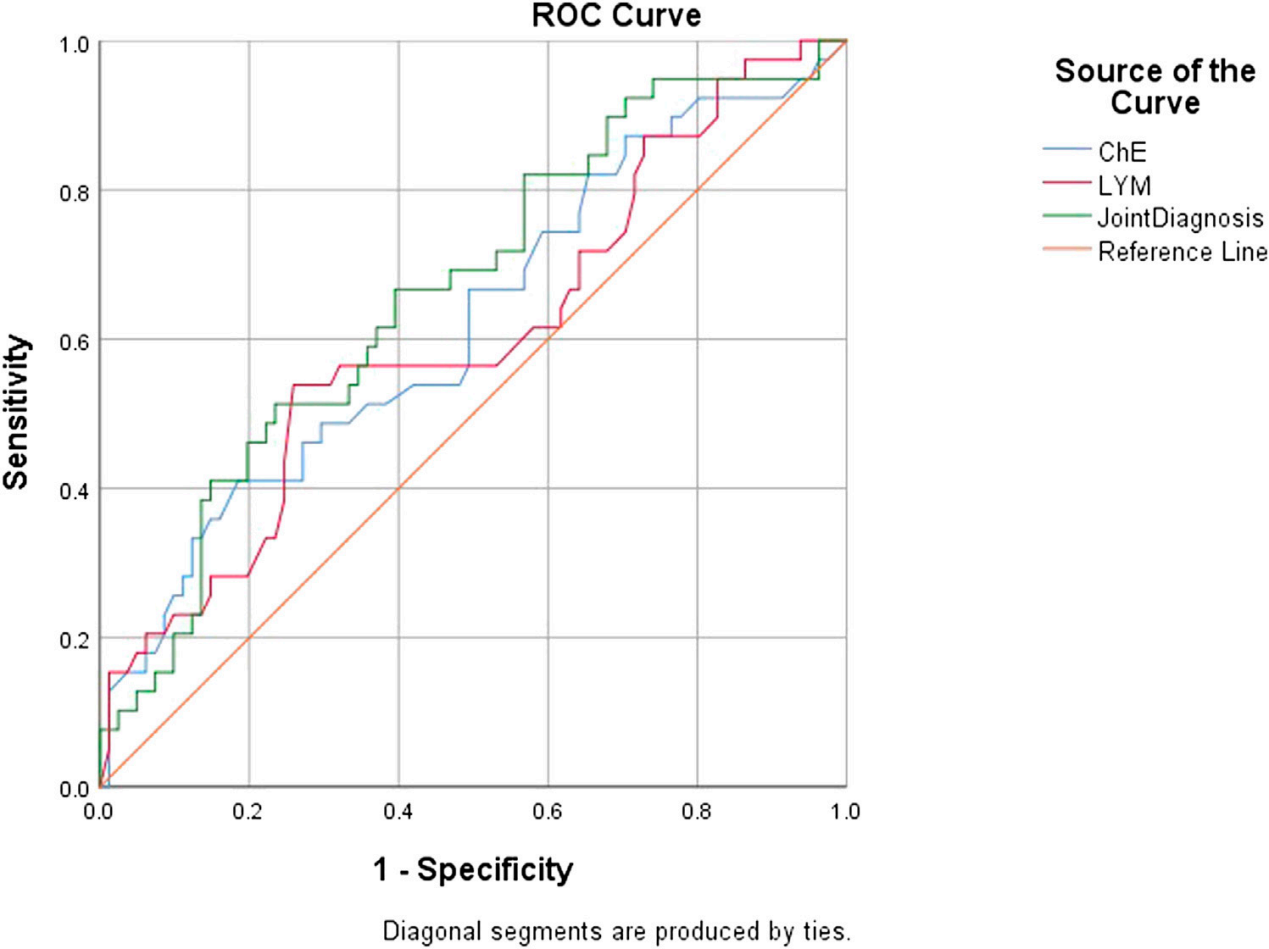
ROC curves of prognostic-related influences of DILI.

The ROC curve analysis showed that the cutoff value of baseline cholinesterase was 4774, with an AUC = 0.621 (95%CI = 0.511–0.730), Youden index 0.225, sensitivity = 0.621, and specificity = 0.815; the baseline lymphocyte cutoff was 1.355, with an AUC = 0.604 (95%CI = 0.493–0.715), Youden index = 0.279, sensitivity = 0.538, and specificity = 0.741. The area under the curve of combined diagnosis was slightly larger than that of single diagnosis, but the specificity and sensitivity of combined diagnosis were lower than those of single diagnosis. Combined diagnosis (AUC = 0.663, 95%CI = 0.560–0.766, *p* = 0.004, sensitivity = 0.513, and specificity = 0.765) (Tables 4, 5) and (Figure 2).

**TABLE 4:** Baseline data multivariate logistic regression analysis.

Variables	*OR*	*p* value	95%*CI*
ChE	0.092	0.059	0.008–1.095
LYM	0.423	0.024	0.201–0.892

**TABLE 5:** Performance analysis of baseline ChE, LYM, and joint diagnosis.

Item	AUC	Sensitivity	Specificity	Cutoff point	Youden’s index
ChE	0.621	0.410	0.815	4774	0.225
LYM	0.604	0.538	0.741	1.355	0.279
Joint diagnosis	0.663	0.513	0.765	0.363	0.278

## 4 Conclusion

Baseline lymphocyte and cholinesterase may be the predictive factors for the chronicity of Chinese herbal medicine–induced liver injury.

## 5 Discussion

Idiotypic drug-induced liver injury (DILI) is a kind of liver injury caused by drugs acting on susceptible people. Unlike inherent DILI, inherent DILI is usually predictable and closely related to drug dosage (Aithal et al., 2011). What is discussed in this study is the idiotypic drug-induced liver injury, hereinafter collectively referred to as DILI. DILI usually presents acute hepatitis or acute cholestasis, and about 10% of patients with DILI develop acute liver failure and may die or require a liver transplant (Ortega-Alonso and Andrade, 2018; Andrade et al., 2019; Stravitz and Lee, 2019).

The incidence of DILI in western countries varies from 1/100000 to 20/100000. The latest research on Chinese mainland shows that the incidence of DILI in Chinese mainland is 23.8/100000 ^1^, which is higher than that in western countries. Traditional Chinese medicine (TCM) or herbal and dietary supplements (HDS) accounted for the highest proportion (26.81%) of the causes of DILI in mainland China, and antituberculosis drugs accounted for 21.99% (Shen et al., 2019). In western countries, antibiotics often account for the highest (Hassan and Fontana, 2019), but in recent years, with the increasing use of HDS, the proportion of DILI caused by HDS in the United States has gradually increased (Maddukuri and Bonkovsky, 2014). TCM and HDS include traditional Chinese medicine, Tibetan medicine, Mongolian medicine, health products with natural medicinal ingredients, herbal medicine, and dietary supplements. TCM and HDS have been more and more widely used all over the world, especially in China, South Korea, and other countries, and a large number of people who take TCM and HDS think that these drugs have less side effects. Also, they take these drugs for different purposes (Shen et al., 2019). Nearly half of them take HDS for health purposes such as getting in shape and losing weight (Maddukuri and Bonkovsky, 2014), while a survey in Hong Kong, China, showed that more than half of cancer patients receiving chemotherapy also take traditional Chinese medicine treatment (Chow et al., 2019). Because many people misunderstand TCM and HDS, the improper use of TCM and HDS has led to liver damage.

Criteria for chronicity of DILI vary from definition to definition^,^ (Benichou, 1990). In this study, the definition of drug-induced liver injury network (DILIN) was used. Chronic DILI is defined as a DILI that persists for at least 6 months with abnormal liver laboratory imaging and histological findings (Fontana et al., 2009). There are different risk factors for the chronicity of DILI; first of all, different drugs have different effects on the chronicity of DILI, and specific drugs such as chlorpromazine and terbinafine can cause acute DILI and, at the same time, also have a higher risk of chronicity (Stine and Chalasani, 2015); the proportion of cardiovascular drugs and central nervous system drugs in chronic DILI is higher than that in all DILI (Andrade et al., 2006; Qiqi et al., 2020). The second factor is the patient himself; studies have shown that older age, women, diabetes, dyslipidemia, and severe DILI are all the risk factors for chronic DILI (Bessone et al., 2019).

Baseline ALT, AST, ChE, and blood routine data were analyzed by binary logistic regression, and it was found that baseline lymphocyte count and baseline cholinesterase after logarithmic transformation were associated with the prognosis of DILI. The results showed that the higher the baseline lymphocyte count and the higher the baseline cholinesterase were, the lower the risk of chronicity was.

Cholinesterase is mainly synthesized by liver cells and released into the blood, which can reflect the synthetic function of liver cells to a certain extent. When liver function is damaged, the synthesis of cholinesterase is reduced, and the activity in the blood is correspondingly reduced (Ogunkeye and Roluga, 2006); the lower the cholinesterase, the more serious the degree of liver injury. Cholinesterase can reflect the synthesis ability of liver more sensitively (Amin et al., 2017). Our study found that the baseline cholinesterase of patients with chronicity of drug-induced liver injury caused by Chinese herbal medicine was lower than that of cured patients, which partly reflected that patients with lower cholinesterase were more likely to develop chronicity.

Lymphocytes include T cells, B cells, and NK cells. The occurrence of drug-induced liver injury is inseparable from the role of lymphocytes (Maria and Victorino, 1997); the drug can be used as hapten after entering the body and can be combined with liver protein on the surface of liver cells to form a drug–protein adduct with immunogenicity and antigenicity and then induce the occurrence of immune response (Tujios and Fontana, 2011). Some drugs can stimulate immune response directly as antigen (Pichler, 2003). The accumulation of lymphocytes in the liver leads to liver injury (El-Ghaiesh et al., 2011). We found that the number of lymphocytes at baseline had a certain impact on the prognosis of patients. Patients with lower baseline lymphocytes were more prone to chronicity. Lower levels of lymphocytes may not cause serious liver injury, but it can also lead to the delay of inflammation and promote the occurrence of chronicity. We did not find similar conclusions in previous related studies. We will design a large sample, multicenter, prospective study to elucidate the mechanism of the effect of cholinesterase and lymphocytes on drug-induced liver chronicity in the future.

The drug-induced liver injury caused by Chinese herbal medicine is uncertain because in areas where Chinese herbal medicine culture is prevalent, many people take unknown drugs without receiving formal medical guidance, which causes different degrees of liver injury, and that is also the reason why the drug information of the patients in this research is unclear. Our results showed no significant difference between herbal medicine and Chinese patent medicine in causing chronic DILI. Our study shows that cholinesterase and lymphocyte count after the occurrence of drug-induced hepatitis have a certain role in predicting the prognosis of hepatitis. However, the AUC of the joint diagnostic factor after combining the two was still below 0.7, failing to reach the desired level, which may be attributed to the sample size. Prospective multicenter studies need to be designed to further validate our findings in the future.

## Data Availability

The original contributions presented in the study are included in the article/Supplementary Files; further inquiries can be directed to the corresponding authors.
